# 我如何诊治阵发性睡眠性血红蛋白尿症

**DOI:** 10.3760/cma.j.cn121090-20251126-00551

**Published:** 2026-01

**Authors:** 蓉 付, 惠 刘

**Affiliations:** 天津医科大学总医院血液内科，天津 300052 Department of Hematology, Tianjin Medical University General Hospital, Tianjin 300052, China

## Abstract

阵发性睡眠性血红蛋白尿症（PNH）是一种罕见的后天获得性造血干细胞克隆性疾病，其临床表现多样，易出现漏诊或误诊。临床应通过多学科协作进行早期筛查。基于精准的临床分型，经典型PNH一线治疗为补体抑制剂，合并骨髓衰竭性疾病者主要针对骨髓衰竭进行治疗，伴有溶血者可应用免疫抑制剂联合补体抑制剂。补体抑制剂治疗过程中需密切监测疗效及并发症。本文结合我院收治的3例典型病例，强调早期筛查的重要性，归纳PNH规范化诊疗要点，对突破性溶血及血管外溶血的诊治提出建议，以提升PNH临床诊疗水平。

## Abstract (en)

Paroxysmal nocturnal hemoglobinuria（PNH）is a rare, acquired clonal disorder of hematopoietic stem cells with diverse clinical manifestations, making it susceptible to missed diagnosis or misdiagnosis. Early clinical screening through multidisciplinary collaboration is therefore essential. Treatment strategies should be guided by precise clinical classification. For patients with classic PNH, complement inhibitors are the first-line treatment, whereas for patients with bone marrow failure, the primary treatment should be focused on the underlying bone marrow failure. In cases accompanied by hemolysis, a combination of immunosuppressants and complement inhibitors can be used. During treatment with complement inhibitors, close monitoring of treatment efficacy and complications is required. Based on three representative cases admitted to our hospital, this article highlights the importance of early screening, summarizes key points for standardized diagnosis and treatment of PNH, and provides recommendations for managing breakthrough hemolysis and extravascular hemolysis, with the aim of improving clinical practices.

阵发性睡眠性血红蛋白尿症（PNH）作为一种后天获得性造血干细胞克隆性疾病，临床主要表现为血管内溶血（IVH）、不同程度的骨髓衰竭和高风险并发血栓等。根据流式细胞术检测结果、网织红细胞计数、血清乳酸脱氢酶（LDH）水平及骨髓细胞学检查，PNH可分为三种临床类型：经典型、伴骨髓衰竭型和亚临床型。笔者结合具体病例探讨如何早期筛查、确诊PNH以及选择合适的治疗方案，供同道参考及借鉴。

一、初诊PNH患者的诊断与治疗

1. 典型病例：例1，女，39岁，2013年于当地医院确诊为再生障碍性贫血（AA），接受环孢素A（CsA）及糖皮质激素治疗1年后血象恢复正常，自行停药。自诉2014年9月感冒后出现深茶色尿，约每年1次，未行诊治。2020年10月患者感冒后出现恶心、腹胀，就诊于我院消化科，腹部B超提示大量腹水，腹部增强CT提示下腔静脉血栓致布-加综合征，予低分子肝素抗凝治疗。2020年11月11日患者行下腔静脉造影术、球囊扩张术、门体静脉分流术及溶栓治疗，11月13日行下腔静脉造影术及球囊扩张术。血常规：HGB 71 g/L，RBC 2.55×10^12^/L，WBC 2.73×10^9^/L，PLT 54×10^9^/L；生化检查：血清LDH 664 U/L，总胆红素（TBIL）108.4 µmol/L，间接胆红素（IBIL）93.4 µmol/L，白蛋白31 g/L，肾功能正常；凝血功能：D-二聚体3 933 ng/ml，半个月后复查全腹增强CT考虑布-加综合征加重，肝淤血较前加重，腹盆腔大量积液较前增多，再次行下腔静脉造影术，可见肝内静脉侧支循环丰富，各引流肝静脉主干未见确切显示，不除外血栓形成。后转诊于血液科，复查血常规：HGB 78 g/L，RBC 2.78×10^12^/L，WBC 5.99×10^9^/L，PLT 68×10^9^/L，网织红细胞百分比（Ret％）4.09％；生化检查：血清LDH 704 U/L，TBIL 105.4 µmol/L，IBIL 90.3 µmol/L；凝血功能：D-二聚体5 436 ng/ml。游离血红蛋白46 mg/L，结合珠蛋白<0.06 g/L，PNH克隆：红系（Ⅱ+Ⅲ型细胞）86.5％，粒系89.89％，Flaer^-^粒细胞97.94％，Flaer^-^单核细胞97.41％，直接抗人球蛋白试验（Coombs试验）阴性，骨髓涂片：增生明显活跃，红系增高，粒系减低，巨核细胞增生；心脏超声：射血分数64％，三尖瓣轻度反流，左室舒张功能减低、收缩功能正常。结合患者临床表现及实验室检查结果，明确诊断为经典型PNH，合并下腔静脉、肝静脉血栓，肝硬化，脾大（4.8 cm×15.6 cm）。因当时国内补体抑制剂尚不可及，故予小剂量激素控制溶血、利伐沙班抗凝、维生素E及碳酸氢钠片治疗。HGB维持在70 g/L左右。2021年2月5日复查腹部增强CT：下腔静脉充盈缺损变化不显著，肝淤血较前略减轻，腹盆腔大量积液较前增多。血管外科、消化内科、血液科及影像科多学科联合会诊，建议予低分子肝素抗凝2周后转为利伐沙班抗凝治疗。患者反复出现月经增多，头晕、乏力明显，于2024年5月18日再次住院，复查血常规：HGB 38 g/L，RBC 1.65×10^12^/L，WBC 1.67×10^9^/L，PLT 44×10^9^/L，Ret％ 3.41％；凝血功能：D-二聚体455 ng/ml；生化检查：LDH 499 U/L，TBIL 104.3 µmol/L，IBIL 97.1 µmol/L。予红细胞输注改善贫血，并予依库珠单抗每周600 mg×4周，随后每2周900 mg治疗，4周后患者脱离输血，并间断口服补铁治疗，24周后复查HGB 123 g/L，RBC 3.64×10^12^/L，WBC 3.5×10^9^/L，PLT 61×10^9^/L，Ret％ 4.5％，D-二聚体260 ng/ml，LDH 190 U/L，TBIL 77.7 µmol/L，IBIL 71.6 µmol/L，复查腹部增强CT提示肝实质强化不均，脾大（4.6 cm×15.0 cm），门体静脉分流术后侧支循环形成，未见腹水征。患者乏力明显改善，已回归正常工作，现依库珠单抗持续应用，未发生溶血及新发血栓。

2. PNH患者需早期筛查、及时诊断：由于PNH为血液系统罕见病，且临床表现多样，异质性强，因此临床中易出现漏诊或误诊。IVH、不同程度的骨髓衰竭和血栓是PNH的主要临床表现，除此之外，还包括疲乏、黄疸与肝脾肿大、肾功能不全、肺动脉高压以及平滑肌功能障碍相关症状（包括吞咽困难、呼吸困难、腹痛、胃胀、背痛、头痛、食管痉挛、勃起功能障碍等）[1]–[2]。据《中国阵发性睡眠性血红蛋白尿症患者生存状况白皮书》[3]数据显示，中国PNH患者平均确诊时间为（22.9±36.5）个月，自发病至确诊的平均就医医院数为3.2个。国内亦有研究结果表明PNH误诊率极高，首次诊断即确诊的患者仅占7％，起病1年内确诊者仅占35.5％[4]–[5]，漏诊、误诊现象普遍。因此《阵发性睡眠性血红蛋白尿症诊断与治疗中国指南（2024年版）》[5]（以下简称指南）特别强化了筛查的必要性。

早期筛查、及时诊断可启动有效治疗，减少并发症的发生，改善生活质量，延长总生存期。指南明确建议的筛查指征如下：①以血红蛋白尿、尿含铁血黄素阳性和（或）血清游离血红蛋白升高为主要表现的IVH。②无法解释的溶血伴铁缺乏、腹痛或食管痉挛、血栓栓塞、血小板减少和（或）白细胞减少。③外周血成熟红细胞Coombs试验阴性、无明显肝脾肿大、极少见红细胞碎片、非感染性溶血性贫血。④骨髓衰竭症：怀疑或确诊的AA或低增生性贫血；或难治性血细胞减少伴一系/多系发育异常；不明原因的血细胞减少症。⑤不同寻常的血栓形成：年轻患者（尤其是45岁以下）；非常见部位血栓形成［如肝静脉（布-加综合征）、其他腹腔内静脉（门静脉、脾静脉等）、海绵窦、皮肤静脉等］；伴有下列征象/疾病的血栓形成：溶血征象、全血细胞减少、抗凝治疗效果欠佳、AA、骨髓增生异常综合征（MDS）。⑥出现如下不能用其他疾病解释的临床表现：呼吸困难、腹痛、头痛、腰痛、吞咽困难、勃起功能障碍、体重减轻等[5]。

3. PNH诊断标准：临床表现符合PNH，实验室检查需完善溶血相关检查，流式细胞术检测锚或锚连蛋白为诊断PNH的金标准，外周血中CD55或CD59阴性中性粒细胞或红细胞>10％（5％～10％为可疑），Flaer阴性中性粒细胞和（或）单核细胞>10％（5％～10％为可疑）可确诊[5]。初诊患者需进行骨髓穿刺涂片及病理以精准分型。此外，还需与后天获得性溶血性疾病相鉴别，包括自身免疫性溶血性贫血（AIHA）、微血管病性溶血性贫血（溶血尿毒综合征、血栓性血小板减少性紫癜）、遗传性多核幼红细胞伴阳性酸溶血试验及感染所致溶血性贫血等。

4. 补体抑制剂治疗：PNH治疗方案的选择基于疾病的精准临床分型：经典型PNH一线治疗为补体抑制剂，粒细胞和（或）单核细胞PNH克隆>10％伴严重IVH［即LDH>1.5倍正常范围上限（ULN）］，同时伴有终末器官损害证据的PNH患者均是启动补体抑制剂治疗的指征[5]–[7]，包括：①严重贫血伴或不伴输血依赖（HGB低于70 g/L或低于100 g/L伴心血管症状）的患者；②与PNH相关的血栓形成（静脉或动脉）；③其他病理原因无法解释的肺动脉高压、肾功能不全、需要应用阿片类镇痛药物治疗的腹痛患者；④PNH合并妊娠患者。亚临床型PNH针对PNH克隆无需特殊治疗，主要针对潜在骨髓衰竭进行治疗。合并其他骨髓衰竭性疾病者建议应用免疫抑制剂联合促造血治疗，若患者PNH克隆比例较高且伴有溶血，可应用免疫抑制剂联合补体抑制剂；有如下指征的年轻患者可考虑行异基因造血干细胞移植治疗：①补体抑制剂治疗失败；②PNH合并重型/难治型骨髓衰竭；③补体抑制剂不可及的严重经典型PNH；④PNH演变的MDS、急性髓系白血病患者。目前国内可及的供一线治疗选择的补体抑制剂是依库珠单抗和伊普可泮。开始用药时应充分告知患者补体抑制剂永久性、持续性治疗的必要性。

依库珠单抗作为远端补体抑制剂的代表性药物，有近20年的临床应用历史。国际PNH登记研究（NCT01374360）（真实世界研究）显示，接受依库珠单抗治疗和从未接受治疗患者的20年预估生存率分别为82％和69％，依库珠单抗治疗高疾病活动度（high disease activity, HDA）患者的获益较非HDA患者更显著[8]。国内外多项临床研究均证实依库珠单抗可减少慢性溶血、输血、血栓等并发症，同时显著改善患者的生活质量，使患者实现生存获益[9]–[12]。依库珠单抗亦可用于儿童、妊娠期及哺乳期患者。

伊普可泮（B因子抑制剂）作用于C5末端通路的上游，不仅可控制PNH的IVH，还可阻止血管外溶血（EVH）。APPOINT-PNH临床研究（纳入初治PNH患者）48周结果显示，患者的平均HGB（122 g/L）持续接近正常水平，疲劳显著改善并长期维持稳定，79.5％的PNH患者HGB≥120 g/L，高达97.5％的患者脱离输血[13]。伊普可泮可同时控制IVH及EVH，且口服给药改善了患者的生活质量及治疗依从性。APPLY-PNH临床研究（纳入经治PNH患者）结果显示，对于既往接受C5抑制剂（依库珠单抗/雷夫利珠单抗）治疗疗效不佳的PNH患者，单药治疗可以获得很好的疗效，且具有良好的耐受性和安全性[14]。APPULSE-PNH临床研究评估了伊普可泮单药口服治疗在接受C5抑制剂治疗、HGB≥100 g/L患者中的疗效和安全性。研究结果显示，大多数患者达到HGB≥120 g/L，疲劳症状及治疗满意度评分均有所改善；所有患者均脱离输血；耐受性良好，无临床突破性溶血（BTH）、主要不良血管事件等报道[15]。伊普可泮已获批用于初治及经治经典型PNH患者的治疗。

此外，雷夫利珠单抗作为第一款长效C5补体抑制剂，可完全且持续抑制C5。美国开展的Ⅲ期、随机、开放标签、活性对照、非劣效性、多中心研究301（NCT02946463）和302（NCT03056040）的结果显示，雷夫利珠单抗的主要研究终点（患者脱离输血和LDH恢复正常的比例）和关键次要研究终点［LDH相对于基线的变化幅度、慢性疾病治疗功能评估（FACIT）疲劳评分较基线的变化、BTH、HGB稳定患者的比例及无血清C5变化］均不劣于依库珠单抗[16]–[17]。目前国内已完成Ⅲ期临床研究，尚未上市使用。

5. 血栓并发症的处理：PNH患者发生静脉血栓的风险较普通人群高62倍[18]，据统计，29％～44％的PNH患者至少发生过1次血栓事件[19]。血栓栓塞（TE）显著增加了PNH患者的死亡风险，是致残和致死的主要原因，在已知死亡原因中占40％～67％[20]，故PNH血栓被称为“恶性程度最高的获得性血栓形成状态”。指南明确提出，对于合并下列情况：未应用补体抑制剂且粒细胞克隆>50％、D-二聚体升高、妊娠、围手术期状况和其他相关血栓危险因素等，如果没有已知的抗凝禁忌证，可酌情在有2种及以上血栓高危因素的患者中应用低分子肝素或华法林启动一级预防[5]。已发生过PNH相关血栓栓塞事件的患者，建议使用补体抑制剂进行二级预防。

一旦诊断PNH合并静脉血栓形成，首先评估出血风险，抗凝治疗期间的严重出血与血小板减少显著相关，对于无抗凝禁忌证的患者，指南建议立即启动补体抑制剂联合抗凝治疗。美国4个中心的真实世界研究显示，21％（267/2 043）的PNH患者发生了TE，接受补体抑制剂治疗的PNH患者TE发生率为21/1 000，明显低于未使用补体抑制剂者（40/1 000），接受和未接受补体抑制剂治疗的患者的2年累积血栓发生率分别为3.9％和18.3％[21]。国际PNH登记研究结果显示，LDH≥1.5×ULN伴有症状的高疾病风险PNH患者中，接受依库珠单抗治疗与IVH减少、主要不良血管事件和血栓发生率降低显著相关[11]。真实世界研究纳入韩国2009年至2020年应用依库珠单抗治疗的80例PNH患者，20例有TE症状的患者中14例TE症状完全缓解[22]。

针对补体抑制剂不可及患者，血栓进展及死亡风险极高，因此建议接受长期抗凝治疗，尽管如此，亦不能完全降低血栓相关风险，危及生命的血栓事件在权衡出血风险后，还需进行溶栓等治疗[23]。

二、EVH的诊断和治疗

1. 典型病例：例2，女，22岁，2012年诊断AA，经CsA、丙种球蛋白等治疗，血象有所恢复，HGB 100 g/L左右，WBC 3×10^9^/L，PLT 90×10^9^/L。患者2020年开始出现尿色加深，伴黄疸，复查血常规HGB 70 g/L，WBC 2.5×10^9^/L，PLT 78×10^9^/L，当地医院查PNH克隆：CD59^-^红细胞73.7％，粒细胞96.9％，单核细胞89.8％，诊断AA-PNH综合征，予泼尼松（0.5～1 mg·kg^−1^·d^−1^）、CsA治疗，间断输注红细胞。2021年5月复查HGB 74 g/L，RBC 2.38×10^12^/L，WBC 5.43×10^9^/L，PLT 98×10^9^/L，Ret％ 6.28％，LDH 1 498 U/L，TBIL 26.6 µmol/L，IBIL 18 µmol/L，入组可伐利单抗（C5抑制剂）临床试验，应用可伐利单抗1 000 mg静脉注射，第1天；340 mg皮下注射，第2、8、15、22天，自第29天开始680 mg皮下注射4周持续治疗。用药24周后HGB 107 g/L，RBC 2.84×10^12^/L，WBC 3.17×10^9^/L，PLT 118×10^9^/L，Ret％ 7.31％，LDH 229 U/L，TBIL 33.5 µmol/L，IBIL 22.5 µmol/L，乏力等症状明显改善。患者持续规律用药，HGB稳定在100 g/L左右。2023年5月（用药后2年）HGB突然下降至63 g/L，Ret％ 16.52％，LDH 442 U/L，TBIL 24.8 µmol/L，IBIL16.9 µmol/L，无感染、劳累、药物、疫苗、手术应激及妊娠等诱因，亦属偶发，无规律，不符合药物效应动力学（PD）或药物代谢动力学（PK）导致的BTH，完善Coombs试验提示C3d阳性，考虑发生EVH。患者因经济原因无法转换为近端补体抑制剂，故在应用可伐利单抗的同时，加用泼尼松1 mg·kg^−1^·d^−1^，1周后HGB恢复至95 g/L，后激素逐渐减停，HGB可稳定在100 g/L，复测Coombs试验仍C3d阳性。患者目前持续可伐利单抗规律治疗，病情稳定，未再发生EVH。

2. EVH的诊断及治疗：在应用末端补体抑制剂过程中，上游补体C3不断蓄积，逐渐结合在成熟红细胞表面，进而巨噬细胞在肝脏、脾脏清除结合了C3的成熟红细胞，从而发生EVH[24]。实验室检查提示HGB明显下降，Ret％升高，IBIL升高，Coombs试验提示C3d阳性，可诊断EVH[24]。在依库珠单抗和雷夫利珠单抗的临床试验中，有临床意义的EVH（cs-EVH）定义为HGB≤95 g/L且网织红细胞绝对计数≥120×10^9^/L，约20％的患者发生cs-EVH（总例数188例）[25]。一项真实世界研究纳入100例接受C5抑制剂治疗的PNH患者，平均治疗时间26.5个月，结果显示仅1例（1/58）患者发生cs-EVH[26]。

若出现补体C5抑制剂相关EVH，可尝试以下治疗方法[5]：①糖皮质激素：参考AIHA的治疗方案，一线推荐1 mg·kg^−1^·d^−1^冲击治疗；②临床试验：尝试其他靶点的补体抑制剂临床试验；③转换为近端补体抑制剂，如伊普可泮、C3抑制剂（国内尚未上市）等；④其他免疫抑制剂：如CsA、西罗莫司等；⑤脾大患者可尝试脾切除，同时应考虑血栓形成问题。

三、BTH的诊断和治疗

1. 典型病例：例3，男，37岁，确诊经典型PNH 7年，间断发作酱油色尿，HGB最低至50 g/L，间断输血，曾口服小剂量激素及中药治疗，2024年5月就诊于我院，血常规：HGB 76 g/L，RBC 2.27×10^12^/L，WBC 3.33×10^9^/L，PLT 152×10^9^/L，Ret％ 5.84％；生化常规：LDH 2 745 U/L，TBIL 56.6 µmol/L，IBIL 45.3 µmol/L，游离血红蛋白606 mg/L，结合珠蛋白<0.06 g/L，PNH克隆：CD59^-^红细胞35.53％，粒细胞93.23％，Flaer^-^粒细胞95.22％，单核细胞90.12％，Coombs试验阴性；骨髓涂片：增生性贫血骨髓象；患者2024年5月开始应用依库珠单抗治疗，24周后复查HGB 88 g/L，RBC 2.16×10^12^/L，WBC 4.96×10^9^/L，PLT 151×10^9^/L，Ret％ 14.45％；生化常规：LDH 382 U/L，TBIL 43.8 µmol/L，IBIL 20.4 µmol/L。患者间断出现呼吸道感染、发热，HGB最低降至63 g/L，Ret％ 14.78％，LDH 453 U/L，TBIL 48.5 µmol/L，IBIL 29.6 µmol/L，间断输血，复查Coombs试验阴性，考虑感染所致BTH。2025年4月患者改为接受伊普可泮治疗，4周后HGB升至133 g/L。目前该患者持续应用伊普可泮治疗中，疾病完全缓解，未发生BTH。

2. BTH的诊断及发生机制：治疗期间患者IVH控制良好后复发，需要考虑是否存在BTH。2024年美国的专家共识提出，HGB较最近一次评估下降>15 g/L，血清LDH再次升高大于1.5×ULN，可诊断BTH[27]。BTH的发生机制涉及PK和PD两种，PK导致的BTH通常较为规律，在下次用药前出现急性溶血发作；而PD导致的BTH往往有一定诱因，无规律，常因感染、疫苗、劳累、手术应激等导致补体系统激活，突破药物的阻断[26]–[27]。根据HGB下降的程度，BTH可分为轻度（HGB下降≥15 g/L且<25 g/L）、中度（HGB下降≥25 g/L且<40 g/L）和重度（HGB下降≥40 g/L）[27]–[28]。根据临床试验数据，依库珠单抗治疗过程中BTH发生率为19.9～21.5次/患者年，雷夫利珠单抗治疗过程中BTH发生率为6.8次/患者年[29]。COMMODORE 3临床试验显示，接受可伐利单抗治疗的PNH患者中3.9％发生过BTH[30]。而伊普可泮的Ⅲ期临床试验显示，BTH的发生率明显低于依库珠单抗和雷夫利珠单抗[31]。

3. BTH的治疗：PK BTH可通过增加药物剂量或缩短用药间隔改善，或转换/联用其他靶点补体抑制剂解决。PD BTH则需密切监测HGB、LDH等实验室检查结果，根据严重程度调整药物剂量和给药频次，并予抗感染、红细胞输注等对症支持治疗。

对于轻度BTH且无临床症状的患者，继续维持原治疗剂量；对于轻度伴溶血相关症状（疲乏、血红蛋白尿、腹痛、呼吸困难、吞咽困难、男性勃起功能障碍等）及中重度BTH患者，建议缩短末端补体抑制剂的给药间隔或提高给药剂量，如依库珠单抗改为10～12 d用药或给药剂量由900 mg增加至1 200 mg，或转换为B因子抑制剂、C3抑制剂等或联合近端补体抑制剂D因子抑制剂[27],[32]–[33]。

2024年《接受治疗的阵发性睡眠性血红蛋白尿症患者中药物动力学突破性溶血的管理专家共识》中[32]，对于择期行疫苗接种或手术、正在接受非每日给药的补体抑制剂治疗患者，建议在给药间隔的早期进行手术或疫苗注射；对于接受每日给药的补体抑制剂患者，则维持原给药方案；对于计划外手术或外伤，正在接受近端补体抑制剂或处于末端补体抑制剂治疗间隔早期的患者，维持原剂量用药；对于处于末端补体抑制剂治疗间隔后期的患者，建议缩短给药间隔；对于正在接受口服补体抑制剂治疗的患者，若超过24 h无法通过胃肠道吸收药物，建议临时改用非口服C5补体抑制剂直至恢复口服药物治疗[32]。

四、补体抑制剂治疗过程中的随访

PNH患者应重视贫血、血栓、感染等相关症状的自我监测，如疲劳、尿色改变、呼吸困难、疼痛、水肿、发热、勃起功能障碍等，发生上述症状时建议及时就医。应用补体抑制剂治疗的过程中，一方面要定期监测临床疗效，复查血常规、网织红细胞计数及百分比、血清LDH和胆红素；补体CH50活性；流式细胞术或Coombs试验检测C5抑制剂治疗后红细胞膜表面C3片段沉积；关注BTH和EVH的发生。另一方面，关注常见并发症，如血栓、肾功能、肺动脉高压等。应用补体抑制剂治疗会增加感染的概率，需接种相关疫苗，同时做好预防感染措施；一旦合并感染，应积极寻找感染灶和病原体，并予针对性抗感染治疗。

综上所述，随着补体抑制剂在国内上市，PNH患者的治疗进入崭新时代。临床应通过多学科协作进行PNH的早期筛查，减少漏诊或误诊，及时启动有效治疗以改善PNH患者的预后。在精准临床分型的基础上选择合适的治疗方案：经典型PNH的一线治疗为补体抑制剂；亚临床型PNH无需针对PNH克隆进行治疗；合并其他骨髓衰竭性疾病者主要针对骨髓衰竭进行治疗，伴有溶血者可应用免疫抑制剂联合补体抑制剂，有合适供者的年轻患者可考虑行造血干细胞移植治疗。补体抑制剂为终身治疗，需密切监测疗效及并发症情况，关注BTH及EVH的发生，必要时可增加剂量、频率或进行补体抑制剂的转换及联合，以期达到最佳疗效。
